# Design of epidermal growth factor immobilization on 3D biocompatible scaffolds to promote tissue repair and regeneration

**DOI:** 10.1038/s41598-021-81905-1

**Published:** 2021-01-29

**Authors:** Teodora Bavaro, Sara Tengattini, Refaya Rezwan, Enrica Chiesa, Caterina Temporini, Rossella Dorati, Gabriella Massolini, Bice Conti, Daniela Ubiali, Marco Terreni

**Affiliations:** 1 Department of Drug Sciences, University of Pavia, Viale Taramelli 12, 27100 Pavia, Italy; 2 Present Address: Department of Pharmacy, ASA University Bangladesh, 23/3 Bir Uttam A.N.M Nuruzzaman Sarak, Dhaka, 1207 Bangladesh

**Keywords:** Biotechnology, Drug discovery, Medical research

## Abstract

Exogenous application of human epidermal growth factor (hEGF) stimulates epidermal wound healing. The aim of this study was to develop bioconjugates based on hEGF mimicking the protein in its native state and thus suitable for tissue engineering applications, in particular for treating skin-related disorders as burns. Ribonuclease A (RNase A) was used to investigate a number of different activated-agarose carriers: cyanogen bromide (CNBr)-activated-agarose and glyoxyl-agarose showed to preserve the appropriate orientation of the protein for receptor binding. EGF was immobilized on these carriers and immobilization yield was evaluated (100% and 12%, respectively). A peptide mapping of unbound protein regions was carried out by LC–MS to take evidence of the residues involved in the immobilization and, consequently, the flexibility and surface accessibility of immobilized EGF. To assess cell proliferative activities, 10, 25, 50, and 100 ng/mL of each immobilized EGF sample were seeded on fibroblast cells and incubated for 24, 48 and 72 h. The immobilized growth factor showed significantly high cell proliferative activity at 50 and 100 ng/mL compared to control and soluble EGF. Although both of the immobilized samples show dose-dependency when seeded with high number of fibroblast cells, CNBr-agarose-EGF showed a significantly high activity at 100 ng/mL and 72 h incubation, compared to glyoxyl-agarose-EGF.

## Introduction

Growth factors (GFs) are soluble, secreted, large polypeptides that can regulate many aspects of cellular functions including proliferation, differentiation, migration, adhesion, and gene expression^1,2^. Local application of growth factors into injures is generally ineffective, since they are either rapidly degraded or washed off from the site of interest^1^.

**Figure 1:**
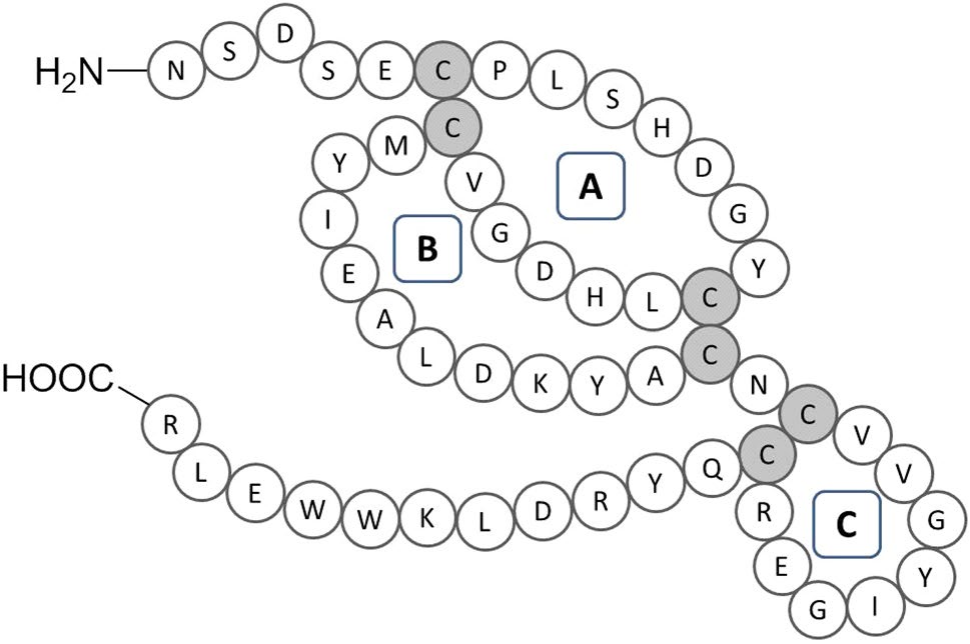
Amino acid sequence of human epidermal growth factor.

Epidermal growth factor (EGF) stimulates the growth of various epidermal and epithelial tissues^3^. Human EGF is a 53 amino acid polypeptide, containing six cysteine residues forming three disulfide bonds, which delimit the A, B and C loops. The A loop goes from residues 6 to 19 and contains α-helix structures, the B loop goes from residues 20 to 31 and forms a two-stranded antiparallel β-sheet, whereas the C loop (residues 33–42) is part of the second antiparallel β-sheet, which are linked by three intramolecular disulfide bonds (Fig. 1)^4,5^. Human EGF has two lysine residues at 28 and 48 positions^6^. EGF plays a vital role in the control of normal dermal wound healing at cellular and molecular level^7^. The recent research interest in EGF due to its crucial role in wound healing shifts the research focus from the investigation of unmodified EGF action in different wound environments to its delivery.

EGF is one of the major ligands of the EGF receptor (EGFR). Members of the EGFR family include ErbB1 (HER-1), ErbB2 (HER-2), ErbB3 (HER-3) and ErbB4 (HER-4)^3^. EGF binds with high affinity to EGFR extracellular region (divided in four domains I-IV) resulting in receptor dimerization and initiation of signal transduction that leads to DNA synthesis and cell proliferation^5^. One EGF binding site in EGFR was identified in domain I, and other two binding sites are in domain III of the extracellular region of the receptor. The receptor domain I interacts mostly hydrophobically with the EGF B loop, involving particularly residues Met21, Ile23 and Leu26. Furthermore, hydrogen bonds between the EGF residues 31–33 and EGFR residues 16–18 form a short parallel β-sheet. The receptor domain III interacts with the A loop of EGF. The hydrophobic interaction between side chains of Tyr13 EGF and Phe357 EGFR residues is important for receptor binding. In addition, Arg41 is an important residue for receptor binding. The *C*-terminal region of EGF binds with the receptor domain III. In particular, Leu47 EGF residue is involved in hydrophobic interactions with Leu382, Phe412 and Ile438, while Gln43 and Arg45 EGF backbone react by hydrogen bonds with Gln384 side chain of EGFR^5,6^.

Cells may respond differently to soluble and immobilized GFs. Covalent immobilization of GFs to biomaterials can be advantageous since soluble GFs are internalized as a substrate-receptor complex, while immobilized GFs are poorly internalized, thus sustaining, by down-regulation of signal transduction inhibition, the signaling pathways inside the cells^7,8^. Immobilization of GFs provides prolonged availability, reduced degradation rate, and increased regeneration efficacy by lowering GF dose and final costs^9^. Immobilized GFs have many applications such as angiogenesis, bone repair and regeneration, dermal wound healing, stem cell differentiation^10^. Several studies showed that immobilized EGF significantly influences signaling, proliferation, migration, and gene expression of keratinocytes and fibroblasts. Recently, biomedical in vivo applications have been developed^11^.

Protein immobilization can be achieved through physical (adsorption or encapsulation) or chemical (covalent or non-covalent) conjugation. Covalent immobilization of GFs to biomaterials is emerging as a promising method for achieving localized and sustained GF delivery^8^. The choice of the most appropriate conjugation method depends on the substrate chemistry, the availability of reactive groups in GF structure, and their involvement in ligand–receptor interactions.

Many immobilization strategies have been developed to improve the stability of EGF for clinical applications. Hardwicke et al*.* immobilized GF on dextrin for application in tissue regeneration. Dextrin-based polymer is biodegradable and increases the stability of GF temporarily masking the protein. In vitro studies showed that EGF, once released, enhanced cellular migration and increased fibroblasts proliferation. Moreover, it improved wound healing after topical application^12^. Kuhl and Griffith-Cima conjugated mouse EGF to aminosilane-modified glass via star poly (ethylene oxide). It was found that conjugated EGF retained its biological activity, and was as effective as soluble EGF^13^. Recently, Masters et al. synthesized 3D scaffolds that were covalently modified with gradients of EGF, in order to create a platform that promotes directed cell migration^14^. Cell migration is a critical element in wound healing, and it is believed that the ability to control the migration direction of cells will lead to accelerated closure of wounds^14,15^. Furthermore, a streptavidin-functionalized scaffold was developed to react with biotinylated EGF, to study breast cancer cell invasion^16^.

An ideal scaffold for protein immobilization must be biocompatible, biodegradable, and biologically inert, it should elicit minimal immune reaction, contain non-toxic degradation products, and it should be porous for ensuring nutrients and gas exchanges. Biomaterials should mimic the dynamic and complex microenvironment of extracellular matrix (ECM), which is essential for tissue regeneration. Natural biomaterials, intended for supporting protein conjugation, should be biocompatible, biodegradable and atoxic. Nevertheless, natural biomaterials are susceptible to enzymatic degradation^17^. Agarose is a natural polysaccharide obtained from marine red algae. It contains repeating units of neoagarobiose, a disaccharide formed by β-d-galactose and 3,6-anhydro-α-l-galactose linked by glycosidic bonds β (1–4). Each neoagarobiose unit is linked by α (1–3) glycosidic bonds forming the agarobiose unit. Each agarobiose unit has four hydroxyl groups; only the primary hydroxyl group exploited for chemical derivatizations^18^. Agarose can mimic ECM in promoting cell adhesion and provides a porous scaffold that permeates nutrients to support cell growth. Agarose and agarose-based biomaterials have been widely used in tissue engineering (TE)^19^.

The aim of this work was to design a bioconjugate based on native hEGF, suitable for the development of new biomedical tools in TE. As a first approach, different immobilization techniques were investigated with the purpose of preferably achieving conjugation between the support and the biomolecule by one-point immobilization through the terminal amino group of the protein, in order to maintain the biological activity of EGF irreversibly linked to agarose. This choice was addressed by the evidence that this site avoids involving amino acids, which are involved in the interaction with the receptor. The conjugation techniques are based on the formation of covalent bonds that prevent biomolecule release from the support achieving a more controlled and targeted effect of hEGF. First, the conjugation of a model protein (ribonuclease A) with differently activated-agarose scaffolds was investigated and two conjugation strategies were selected according to their selectivity for the terminal amino group: the covalent binding with CNBr-activated-agarose and glyoxyl-agarose scaffold. To take evidence of amino acids involved in the covalent binding to the support, a previously developed LC–MS/MS method was applied^20^. The immobilized proteins were digested with chymotrypsin and released peptides, corresponding to the unbound areas of the supported protein, were analyzed by LC–ESI–MS/MS. Then, the immobilization of hEGF by selective covalent binding with the prepared scaffolds was carried out. The peptide mapping of the immobilized protein allowed an experimental correlation between the binding chemistry used and the conformational freedom and accessibility of the epitopes responsible of the biological activity. Finally, the activity of the immobilized EGF was investigated on fibroblast cells by monitoring cell attachment and viability.

## Materials and methods

### Materials

All chemicals were of analytical or reagent grade and were used without further purification, unless stated otherwise. 6% Crosslinked agarose was purchased from ABT (Madrid, Spain). Cyanogen bromide-activated-SEPHAROSE-4B (CNBr-agarose), 3-aminopropyl-triethoxysilane (APTS), *N*,*N*’-carbonyldiimidazole (CDI), 3-glycidyloxypropyl-trimethoxysilane (GPTS), bovine serum albumin (BSA), Bradford reagent, ribonuclease A from bovine pancreas (RNase A), glycidol, sodium periodate, sodium borohydride were purchased from Sigma-Aldrich Srl (Milan, Italy). Potassium iodide was purchased from Aldrich-Chemie (Taufkirchen, Germany). Sodium hydrogen carbonate was purchased from Carlo Erba Reagents Srl (Rodano, Italy); potassium di-hydrogen phosphate was purchased from AppliChem Panreac, ITW Companies (Milan, Italy). Tris hydrochloride was purchased from MP Biomedicals (Eschwege, Germany). Epidermal Growth Factor (EGF) was purchased from Twin Helix (Milan, Italy). Materials used to perform SDS-PAGE electrophoresis were purchased by Thermo Fisher Scientific, Life Technologies Italia (Monza, Italy): SureLock Retrofit Kit for XCELL II Mini-Cell (INVITROGEN); NuPAGE Novex 16% Tricine Gel 1.0 mm, 12 Well (INVITROGEN); NuPAGE NOVEX Tricine SDS Running Buffer (INVITROGEN); NOVEX Tricine SDS Sample Buffer (INVITROGEN); SILVERQUEST Staining Kit (INVITROGEN); low Range Protein Ladder (THERMO SCIENTIFIC). The bands were visualized by BioRad ChemiDoc system using ImageLab software. LC–MS/MS analysis was performed on a Dionex Ultimate 3000 RSLC System coupled with a LTQ (Thermo Fisher, Milan, Italy) mass spectrometer equipped with an electrospray ionization source (ESI). Column: Acclaim PepMap100 C18 (3 μm, 100 Å, 300 μm I.D. × 1.5 cm). Normal human dermal fibroblasts (NHDFs) cells were obtained from Promocell (Milan, Italy). Dulbecco's Modified Eagle's Medium (DMEM), DMSO (dimethyl sulfoxide), 3-(4,5-dimethylthiazol-2-yl)-2,5-diphenyltetrazolium bromide (MTT), trypsin-ethylenediaminetetraacetic acid, penicillin/streptomycin were purchased from Sigma Aldrich (Milan, Italy). DAPI and phalloidin staining were obtained by Sigma Aldrich (Milan, Italy).

### Immobilization of RNase A

RNase A immobilization was achieved by applying the immobilization procedures reported below. The immobilization reaction was monitored by Bradford assay, by measuring the amount of protein in the supernatant before and after addition of the carrier at different endpoints^21^. The immobilization mixture was filtered, washed with distilled water and dried under vacuum. The immobilization yield (%) was calculated according to the following Eq. (1):

1 $${\text{Yield}}\;(\% ) = \frac{{{\text{immobilized}}\;{\text{protein}}}}{{{\text{starting}}\;{\text{protein}}}} \times 100$$

### Immobilization of RNase A on CNBr-agarose

Commercial CNBr-agarose (250 mg) was treated with 1 mM hydrochloric acid (50 mL) under mild mechanical stirring for 30 min. The mixture was then filtered, washed with distilled water, and dried under vacuum. RNase A (500 μg) was placed into the immobilization vessel containing 3.15 mL of potassium phosphate buffer (50 mM, pH 7.0) and CNBr-agarose was added thereto. Immobilization was carried out under mechanical stirring for 4 h at room temperature. The immobilization reaction was quenched at 4 °C in 40 mL of 0.1 M TRIS–HCl buffer, pH 7.4, by keeping the mixture under stirring for 2 h. The suspension was recovered by filtration and washed with ultrapure water, dried under vacuum and stored at 4 °C.

### Immobilization of RNase A on glyoxyl-agarose

Glyoxyl-agarose was prepared according to Bavaro et al.^22,23^. RNase A (500 μg) was poured into the immobilization vessel containing 3.15 mL of 50 mM potassium phosphate buffer (pH 7.0). Glyoxyl-agarose (250 mg) was added to the vessel. Immobilization was carried out for 4 h under mechanical stirring. Then, chemical reduction of the Schiff bases was carried out by adding 14 mg of NaBH_4_ (1 mg/mL) over 30 min. The suspension was filtered, and the immobilized protein was washed with ultrapure water, dried under vacuum and stored at 4 °C.

### Immobilization of RNase A on glutaraldehyde-agarose

The glutaraldehyde-activated agarose was prepared as previously reported^22,23^. Briefly, glyoxyl-agarose (17.5 g) was reacted with ethylendiamine (2 M EDA, pH 10.0) for 2 h, and reduced with NaBH_4_ (1 g) for further 2 h. The EDA-activated agarose was then suspended in 0.2 M phosphate buffer pH 7.0 (3.4 mL) and a solution of 25% (v/v) glutaraldehyde (5.1 mL) was added. The mixture was kept under stirring for 16 h, at room temperature protected from light. Agarose-glutaraldehyde (1 g) was suspended in 50 mM potassium phosphate buffer pH 7.0. RNase A (20 mg) was added and the suspension (14 mL) was kept under mild mechanical stirring for 3 h. Chemical reduction of the Schiff bases was carried out by adding 14 mg of NaBH_4_ (1 mg/mL) to the mixture over 30 min. The immobilized enzyme was then filtered and washed with 10 mM potassium phosphate buffer (pH 7.0), dried under vacuum and stored at 4 °C.

### Immobilization of RNase A on APTS-glutaraldehyde-agarose

RNase A was immobilized by modifying a previously reported protocol^24^. Agarose (3 g) was suspended in 50 mL of 0.5 M NaOH solution for 2 h, filtered and washed with ultrapure water. The carrier was activated in 60 mL solution of 3-aminopropyl-triethoxysilane (APTS, 10% v/v in water) for 24 h and filtered. The agarose-APTS (2.5 g) was suspended in 10 mL of 200 mM potassium phosphate buffer (pH 7.0) and 25% (v/v) glutaraldehyde (6 mL) was added. The mixture was kept under stirring for 16 h at room temperature protected from light. The agarose-APTS-glutaraldehyde (1 g) was suspended in 50 mM potassium phosphate buffer pH 7.0. After the addition of RNase A (20 mg), the suspension (14 mL) was kept under mechanical stirring for 24 h. Chemical reduction of the Schiff bases was carried out by adding 14 mg of NaBH_4_ (1 mg/mL) over 30 min. The immobilized enzyme was then filtered and washed with 10 mM potassium phosphate buffer pH 7.0, dried under vacuum and stored at 4 °C.

### Immobilization of RNase A on CDI-agarose

RNase A was immobilized by modifying a previously reported protocol^25^. Agarose (3 g) was suspended in 50 mL of dry acetone and 5 g of 1,1′-carbonyldiimidazole (CDI) was added. The mixture was kept under stirring overnight at room temperature. The agarose-CDI was filtered, washed twice with dry acetone to remove excess CDI and then dried at 50 °C for 3 h. The agarose-CDI (1 g) was suspended in 50 mM potassium phosphate buffer pH 7.0. After the addition of RNase A (20 mg), the suspension (14 mL) was kept under mechanical stirring during 24 h. The immobilized enzyme was then filtered, washed with 10 mM potassium phosphate buffer pH 7.0 and stored at 4 °C.

### Immobilization of RNase A on GPTS-agarose

RNase A was immobilized by modifying a previously reported protocol^26^. Dry agarose (3 g) was suspended in 60 mL of dry toluene. 3-Glycidyloxypropyltrimethoxysilane (GPTS, 3 mL) and triethylamine (0.45 mL) were added. The mixture was kept under stirring for 24 h at room temperature. Then, the agarose-GPTS was filtered and washed twice with dry toluene to remove excess GPTS and then dried at 50 °C for 3 h. The agarose-GPTS (1 g) was suspended in 100 mM potassium phosphate buffer pH 7.0. After the addition of RNase A (20 mg), the suspension (14 mL) was kept under mechanical stirring for 24 h. The immobilized enzyme was then filtered and washed with 10 mM potassium phosphate buffer pH 7.0, dried under vacuum and stored at 4 °C.

### Immobilization of EGF

Human EGF was immobilized on CNBr-activated-agarose and glyoxyl-agarose. For immobilization, a loading of 2 mg of EGF per g of carrier was performed at pH 7.0 with the procedure established for RNase A immobilization (immobilization time 4 h). Thus, the procedures above described were followed to prepare and activate carriers and immobilized EGF to obtain the CNBr-EGF and the Glyoxyl-EGF7 derivatives. Immobilization of EGF on glyoxyl activated-agarose was also performed at pH 10.0 (Glyoxyl-EGF10). The immobilization yield (%) was calculated according to the paragraph above reported.

### Polyacrylamide gel electrophoresis

Each sample of the EGF immobilization reaction supernatant (20 μL) was transferred into a 1.5 mL EPPENDORF tube containing 20 μL of Novex Tricine SDS sample buffer and mixed. The samples were heated (80 °C) for 5 min in a water bath. A pre-cast 16% Tricine gel was inserted into the electrophoresis chamber and running buffer was poured into the chamber. Each sample (20 μL) and protein ladder were loaded on the gel. The electrophoresis run was performed at 120 V for 2 h using a BioRad electrophoresis power supply. The gel was removed and stained by silver staining (Silver Quest Staining kit protocol, Thermo Fisher). The stained gel was placed in a white light conversion screen (BioRad) and imaged using ChemiDoc XRS + Imaging system (BioRad). The amount of protein in the gel was analyzed by the software ImageLab (BioRad) as not immobilized EGF percentage.

### Chymotryptic digestion of in-solution and immobilized RNase A and EGF

Chymotryptic digestion of in-solution and immobilized proteins was performed according to the literature^20,27^. Briefly, 100 μL of protein aqueous solution (100 μM) was mixed with 90 μL of ammonium bicarbonate (100 mM, pH 8.0), and 10 μL of 100 mM DTT solution in 100 mM ammonium bicarbonate, pH 8.0 for in-solution digestion of RNase A and EGF. The solution was first heated at 60 °C for 30 min for disulfide bridge reduction, and then added of chymotrypsin to a final protein/enzyme ratio of 100:1 (w/w). Then, the solution was incubated overnight at 37 °C under continuous stirring. The reaction was stopped by adding 2.5% (v/v) TFA. For the chymotryptic digestion of immobilized RNase A and EGF, 25 mg of immobilized protein were suspended in 1 mL of ammonium bicarbonate (100 mM), pH 8.0. 50 µL of DTT (100 mM) were added to the suspension, and then kept under stirring for 30 min at 60 °C to reduce disulfide bonds. After conditioning at 37 °C, chymotrypsin was added in order to have a protein/enzyme ratio of 20:1 (w/w). The suspension was incubated at 37 °C under stirring. After 12 h, a second aliquot of chymotrypsin was added. Digestion was stopped by adding 20 µL of TFA, the mixture was recovered by centrifugation at 112 g for 10 min. Afterwards the supernatant was withdrawn, filtered and subjected to reverse phase liquid chromatography-tandem mass spectrometry (RP-LC–MS/MS) analysis.

### RP-LC–MS/MS analysis of digestion mixtures

Peptides released by chymotryptic digestions were analyzed by capillary RP-ESI–MS/MS, by applying a previously developed method^28^. Briefly, the chromatographic separation was performed on a Dionex UltiMate 3000 HPLC system (Thermo Fisher Scientific, Waltham, MA, USA) equipped with an UltiMate 3000 Autosampler and an UltiMate 3000 Variable Wavelength Detector and controlled by Chromeleon software (version 6.8). The analytical column was a C18 ACCLAIM PepMap RSLC (300 μm × 150 mm, 2 μm, 100 Å) from Dionex (Thermo Fisher Scientific, Waltham, MA, USA) and the mobile phases were water + 0.05% TFA (v/v) (solvent A) and water/ACN 20/80 + 0.04% TFA (all v/v) (solvent B). The gradient was from 4 to 55% B in 40 min with a flow rate of 4 μL min^−1^. The volume of sample loaded was set at 1 µL and column compartment temperature at 30 °C. MS detection was carried out on a LTQ-MS with an ESI source controlled by X-calibur software 2.0.7 (Thermo Fisher Scientific, Waltham, MA, USA), under the following constant instrument conditions: positive ion mode, source voltage 4.5 kV, capillary voltage 31 V, sheath gas flow rate 40 (arbitrary units), auxiliary gas flow rate 10 (arbitrary units), capillary temperature 250 °C, tube lens voltage 95 V. Full scan mass range was fixed from 300 to 2000 Da. MS/MS spectra were obtained by collision induced dissociation (CID) with normalized collision energy of 350. Data processing was performed using Bioworks Browser (Thermo Fisher Scientific, revision 3.1) by comparing experimental data against the protein database sequences (*.fasta) using SEQUEST algorithm. The *.fasta format for RNase A and EGF were downloaded from Expasy (P61823 and P01133, position 971–1023, respectively). Peptides selection was achieved using SEQUEST filtering criteria Xcorr score > 1.0. All the identified spectra were manually confirmed to avoid false positive results.

### Cell culture

Fibroblasts of adult human dermis derived from primary culture (NHDF, Normal Human Dermal Fibroblast) were maintained at 37 °C and 5% CO_2_, in DMEM containing 10% v/v of fetal bovine serum (FBS) and 1% v/v of antibiotic mixture (100 µg/mL penicillin, 100 µg/mL streptomycin). At the confluence, the fibroblasts were subjected to trypsinization and resuspended in suitable volume of DMEM to continue with cell expansion until passage 5 was reached.

### In vitro cytotoxicity study

The cytotoxicity study on immobilized and free EGF was carried out on NHDF cell line to evaluate whether the immobilization of EGF on glyoxyl-agarose and CNBr-activated-agarose carriers have an effect on the in vitro cell viability. The effects of immobilized EGF on cell viability were assessed using 96 well cell culture cluster with 10,000 fibroblasts plated in contact to different amounts of immobilized EGF concentrations ranging from 10 to 100 ng/mL (10, 25, 50 and 100 ng/mL). The cell viability was evaluated after 24 and 48 h by a microculture tetrazolium (MTT) assay. The results were expressed as the absorbance at 570 nm, and compared with EGF.

### Cell viability study

Outcomes of soluble-EGF and immobilized-EGF on promoting cell-growth were tested on NHDF (5000 cells/well). Cells were cultured in DMEM w/ FBS for 24 h, following medium was discarded and replaced with DMEM w/o FBS. Cells were incubated for 7 days with 50 ng/mL of each sample, as free EGF, immobilized EGF and glyoxyl-agarose and CNBr-activated-agarose carriers. At scheduled endpoints (3, 5 and 7 days), cells were washed with PBS before to be incubated for 3 h with MTT solution, (5 mg/mL in DMEM w/o FBS). Purple formazan crystals were dissolved in DMSO, under mild stirring at room temperature for 45 min; absorbance (Abs) was determined by microplate reader ELx800, BioTek at a wavelength of 570 nm. Cell viability data were tabled as percentage of viable cells towards untreated cells (CRT), (n = 3).

### Fluorescent staining and analysis

NHDF cells grown on coverslips were treated with 50 ng/mL of soluble EGF, immobilized-EGF and glyoxyl-agarose and CNBr-agarose carriers for different endpoints (3, 5 and 7 days) and then fixed with freshly prepared 4% w/v para-formaldehyde for 10 min at room temperature and tilted agitation (8 osc/min). Following, they were mildly washed with PBS for 10 min at room temperature. In order to increase permeability, ethanol 70% v/v was added to the coverslips and maintained at − 4 °C, overnight. Phalloidin (10 nM) was dissolved in methanol and used as stock solution. 5 µL of stock solution was diluted into 245 µL di PBS, the volume added to each sample was 20 µL, and then incubated in dark for 20 min. The coverslip was washed with PBS and following 500 µL DAPI solution (800 nM) was supplemented, the sample was washed with PBS before microscope analysis.

### Statistical analysis

Biological results were tabled as mean ± SD, (n = 3). Two-way ANOVA followed by Tukey's multiple comparison test were used for mean values differences analysis between experimental groups. Probability values less than 0.05 (*p < 0.05) were set out as significant, while 0.01 (**p < 0.01), 0.0001 (****p < 0.0001) as highly significant.

## Results and discussion

The aim of this study was to immobilize EGF exploiting an immobilization chemistry which enables the exposure of EGF active surface necessary to preserve/enhance its functionality. The amino acid sequence of EGF is composed of 53 amino acids and contains two Lys (K) residues. As most of the immobilization chemistries involves the amino group of Lys side chain, these two residues may be involved in the interaction with the carrier, restricting the conformation flexibility of the protein. The lack of conformation freedom, as well as the steric hindrance arising from immobilization of the protein with the sequences responsible of the biological activity oriented toward the scaffold surface may negatively affect the biological activity of the immobilized EGF. The use of *N*-terminal amino acid residue targeted immobilization strategies would be the ideal solution to prepare an active and flexible supported EGF. In addition, EGF has three disulfide bridges that should be preserved during immobilization.

### Immobilization of the model protein (RNase A) on different carriers

For a detailed study of the selectivity of a number of immobilization strategies and supports, RNase A was used as a model protein because of the high number of Lys residues on the surface of this protein that can be involved in the immobilization process. In fact, RNase A has 124 amino acids (13.7 kDa), four disulfide bridges and 10 Lys residues, which enabled a fine tuning of the protein orientation and a more reliable rationalization of the effect of immobilization/support on protein surface reactivity. Before chemically immobilizing RNase A onto the scaffold, a spacer molecule may be utilized to allow the immobilized biomolecule to move into a suitable orientation and to function properly, overcoming the steric effects between the functional groups on the scaffold surface and those of the biomolecule. In order to find the best covalent immobilization approach in terms of selectivity for the terminal amino group, agarose was functionalized with different reactive groups: aldehyde, glutaraldehyde, 3-glycidoxypropyltrimethoxysilane (GPTS), 3-aminopropyltriethoxysilano (ATPS)-glutaraldehyde, *N*,*N*′-carbonyldiimidazole (CDI) and cyanogen bromide (CNBr). A total of six RNase A-bioconjugates were obtained (Table 1).

**Table 1:** Immobilization of RNase A on different supports.

Sample	Support	Activation	Yield (mg of protein/mg support)* (%)
1	Glyoxyl-agarose	Aldehyde	36
2	CDI-agarose	Carbamate	40
3	GPTS-agarose	Epoxy	99
4	Glutaraldehyde-agarose	Aldehyde	95
5	APTS-glutaraldehyde-agarose	Aldehyde	40
6	CNBr-agarose	Isocyanate	65

*Bradford Assay. Loading: 20 mg RNase A/g support.

*APTS *(3-aminopropyl) triethoxysilane, *CDI N*,*N*′-carbonyldiimidazole, *GPTS *(3-Glycidyloxypropyl) trimethoxysilane, *CNBr *cyanogen bromide.

Agarose activated with aldehyde groups (glyoxyl-agarose) is routinely used as a hydrophilic carrier for immobilization and stabilization of enzymes. Glyoxyl-agarose is not very efficient in immobilizing proteins at neutral pH values, because only in some exceptional cases there are enough amino groups in the proteins that are reactive under these conditions (e.g., multimeric enzymes, some proteolyzed proteins) to permit the first bond. However, if the immobilization of the proteins at neutral pH values is performed in the presence of a Schiff’s base reducing or stabilizing agent it is possible to get acceptable protein immobilization yields^29,30^. Hence, with the aim to avoid multipoint immobilization and to drive the protein towards one-point attachment, a neutral pH was used. The immobilization yield of RNase A after 3 h was 36% (Sample 1, Table 1). Sodium borohydride was added as reducing agent to quench the unbound aldehyde groups of the carrier and turn them into hydroxyl groups. Different immobilization methods were also investigated in order to obtain covalent immobilization of RNase A avoiding the reduction step. To this purpose, CDI was used to activate agarose. CDI is a highly reactive carboxylating agent that contains two acylimidazole leaving groups forming reactive carbonyl groups on the hydroxyl support. Briefly, CDI was exploited to convert agarose primary hydroxyl groups in imidazolylcarbamate groups, which react with primary amine of the RNase A forming a carbamate. Activation step was performed in anhydrous media since CDI is susceptible to hydrolysis. The bioconjugate was obtained in 40% yield, after 24 h (Sample 2, Table 1). RNase A immobilization on GPTS-agarose has the advantage of inserting a longer spacer arm, which allows to decrease protein constrictions at surface and provide higher flexibility. Coupling reaction was carried out for 24 h to allow the correct alignment of the reactive groups^29^. The bioconjugate was obtained in a quantitative yield (Sample 3, Table 1). RNase A immobilization on glutaraldehyde-agarose introduces a spacer between the protein and the carrier surface. Immobilization occurs through the formation of Schiff’s bases generated from the reaction between the RNase A amino groups and the aldehyde groups of the spacer. The immobilization yield of RNase A bioconjugate was 95% (Sample 4, Table 1).

**Figure 2:**
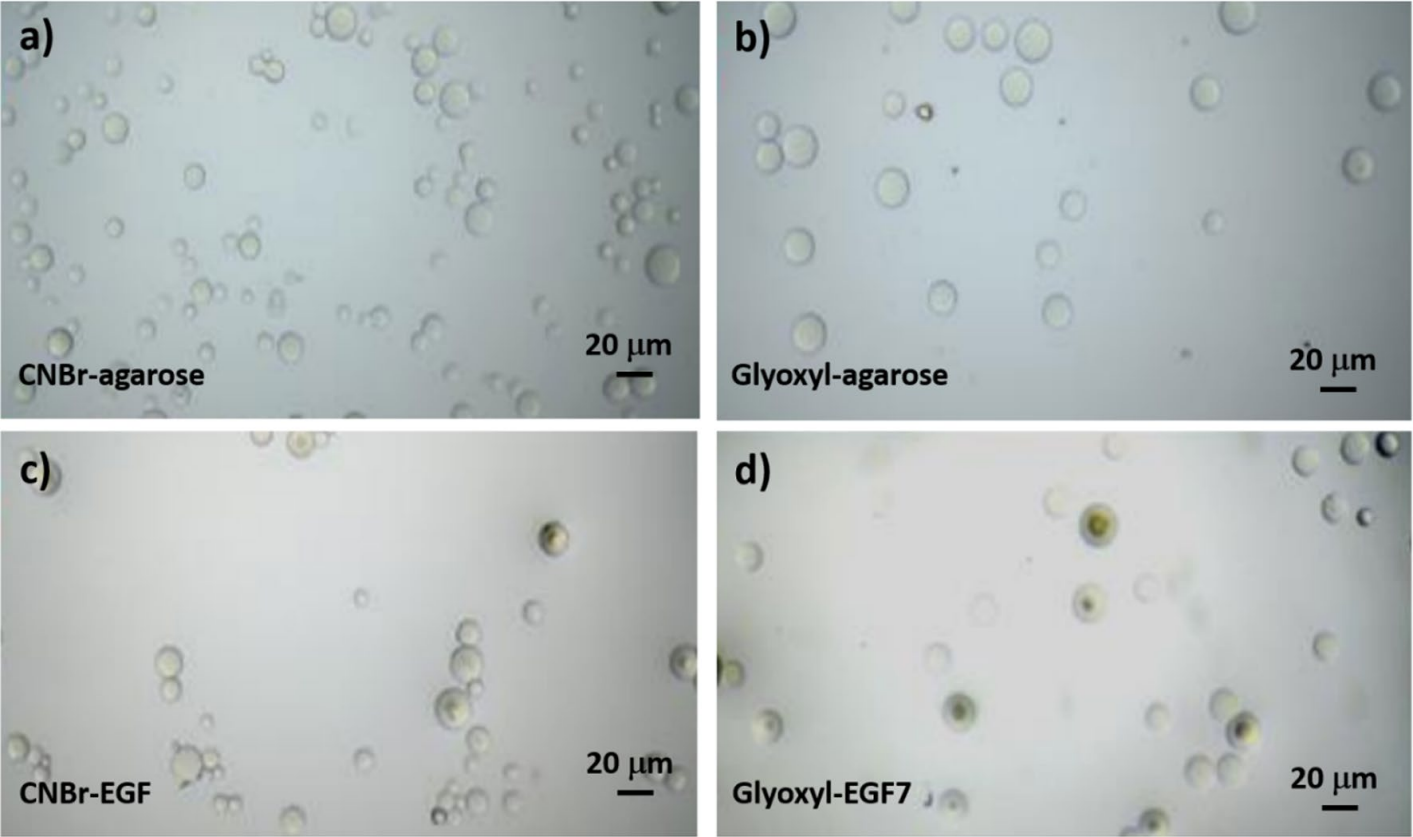
Microscopic images of CNBr-activated-agarose and glyoxyl-agarose carriers before (**a**,**b**) and after EGF immobilization (**c**,**d**), scale: × 40. For each sample n = 30.

APTS glutaraldehyde-agarose and glutaraldehyde-agarose rely on a similar binding chemistry with RNase A. Agarose was activated using APTS as a coupling reagent forming Schiff’s bases by nucleophilic attack of RNase A NH_2_ terminus group turning into an increased distance between the protein and the carrier. The bioconjugate was obtained in 40% yield after 24 h (Sample 5, Table 1). CNBr activated-agarose is more commonly employed to bind covalently biomolecules to active carriers. CNBr reacts with hydroxyl groups on agarose to form cyanate esters or imidocarbonates. These groups readily react with primary amines under very mild conditions; the clear result is the covalent coupling of ligand to the agarose matrix. The preferred resultant structure is an imidocarbonate, which has no net charge. The bioconjugate was obtained in 65% yield after 3 h (Sample 6, Table 1). In Fig. 2 optical microscopic images of CNBr-activated-agarose and glyoxyl-agarose carriers before and after EGF immobilization. Agarose carriers are spherical in shape and with a relatively homogeneous size distribution. The mean size of CNBr-agarose and glyoxyl-agarose are 16.78 ± 2.845 μm and 16.089 ± 3.246 μm while their EGF immobilized counterparts are 12.42 ± 3.75 μm and 12.398 ± 3.939 μm, respectively.

### Peptide mapping of immobilized RNase A: protein orientation assessment on the solid support

**Figure 3:**
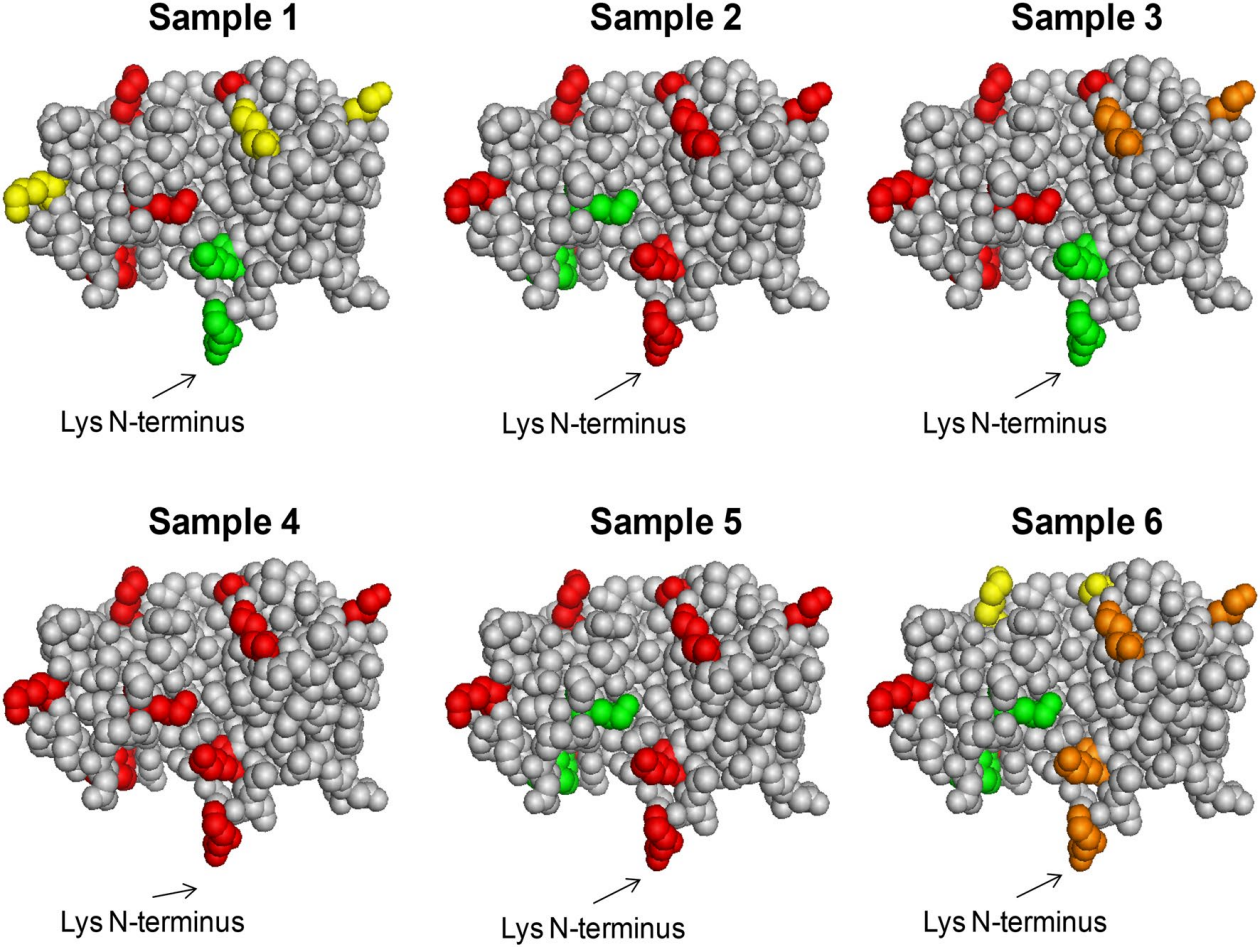
Analytical characterization of Samples 1–6. Relative abundance of Lys residues obtained by peptide mapping of free amino acids in RNase A conjugated with different scaffolds: glyoxyl-agarose (sample 1), CDI-agarose (sample 2), GPTS-agarose (sample 3), glutaraldehyde-agarose (sample 4), APTS-glutaraldehyde-agarose (sample 5) and CNBr-agarose (sample 6). Colors indicate the percentage abundance of each Lys residue normalized respect to the most abundant peptide in the corresponding analysis. The following legend was used: red 0–20%; orange 20–40%; yellow 40–60%; green 60–100%. Consequently, red = Lys bound to the carrier; green = Lys exposed on the surface, not involved in the binding. Created with PyMOL Molecular Graphics System (version 1.1 eval, http://www.pymol.org).

To assess the orientation of immobilized RNase A, a strategy developed by some of the authors has been applied^20^ in order to define the amino acids of RNase A involved in the immobilization performed on different activated-supports. The immobilized protein was submitted to hydrolysis with chymotrypsin and the released peptides analyzed by capillary LC–ESI–MS/MS analysis. Chymotrypsin should cleave the most accessible areas of the immobilized protein, resulting in the release and subsequent detection of peptides that are not directly involved in the chemical binding with the support. On the contrary, peptides involved in the immobilization are not accessible to enzyme cleavage and they will be missing in the LC–ESI–MS trace. The peptide map obtained from the LC–MS/MS analysis allows to deduce both qualitative and semi-quantitative information on protein orientation when immobilized on a carrier, given the amino acid sequence and the three-dimensional structure (conformation) of RNAse A. At first, soluble RNase A was digested by chymotrypsin to list and characterize all the generated peptides (Table S1, Supplementary Information). A sequence coverage of 94% was obtained. Then, all the RNase A bioconjugates listed in Table 1 were submitted to digestion and LC–MS/MS analysis as above described. Each digestion and analysis was performed in duplicate. The peptides identified in each sample are reported in Table S1. The mass area of each peptide containing one or more Lys normalized to the mass area of the most abundant peptide in the trace was considered as a semi-quantitative index of involvement of such amino acids in the binding with the carrier. In Fig. 3, Lys of RNase are highlighted according to their abundance as free amino acid. Red colored Lys represent the residues highly involved in the binding to the carrier, while green residues represent those Lys not covalently bound to the matrix, which are all exposed on the protein surface. For samples 2 and 5 (Table 1) almost all the Lys residues were involved, except two, namely K36 and K41 (Fig. 3). In Sample 4, no free K were found in the digested solution, revealing an extensive multipoint covalent attachment using this immobilization approach. The NH_2_ terminus position of RNase A was totally excluded from linkage to the carrier in Samples 1 and 3, with all the remaining residues being involved at a different extent. Although in the Sample 1 RNase A was not bound to the matrix by Lys *N*-terminus, few interactions occurred between the protein and the matrix. Sample 6 was the only one suggesting the main binding through the *N*-terminus and only partial involvement of surrounding residues.

Based on these results, glyoxyl-agarose-RNase A and CNBr-agarose-RNase A bioconjugates (Sample 1 and 6) could show an adequate orientation and accessibility to EGF receptor. Accordingly, these immobilization methods were selected to immobilize EGF. Before this, RNase A was immobilized on both these carriers by using a lower protein loading per g of carrier (2 mg RNase A/g of carrier). By using this protein loading, RNase A immobilization on CNBr-activated-agarose gave a quantitative yield (100%) in less than 3 h, while the immobilization yield of RNase A on glyoxyl-agarose was 31% after 5 h.

### Immobilization of EGF

**Figure 4:**
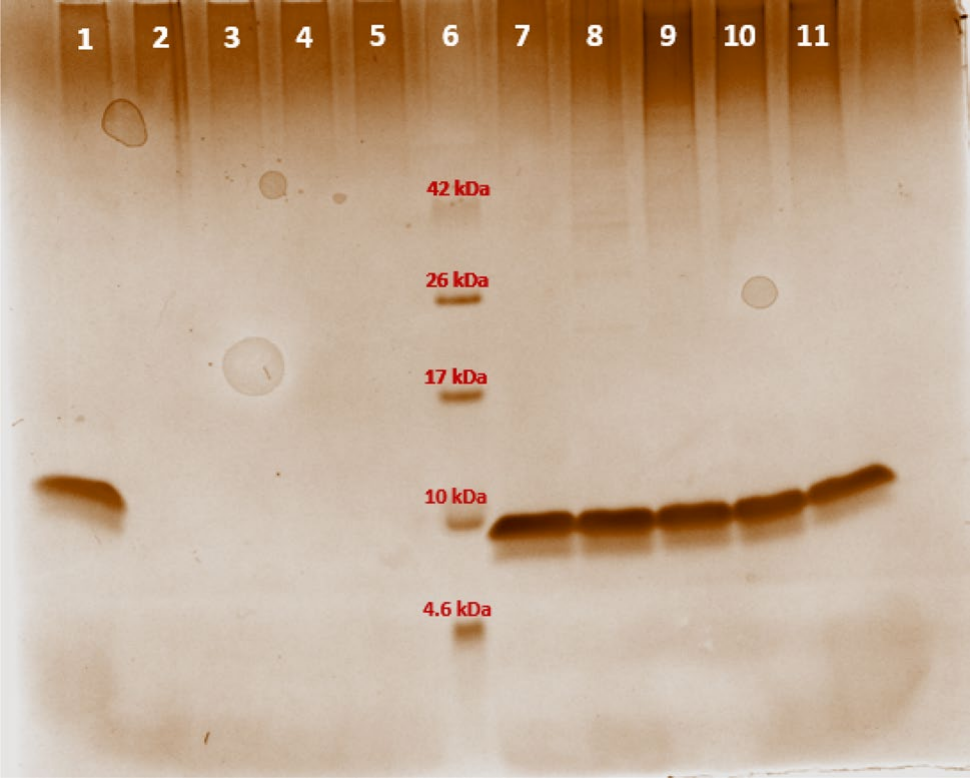
Monitoring EGF immobilization by polyacrylamide gel electrophoresis. Lane 1. 100% CNBr-EGF, 0 h; Lane 2. 0% CNBr-EGF, 1 h; Lane 3. 0% CNBr-EGF, 2 h; Lane 4. 0% CNBr-EGF, 3 h; Lane 5. 0% CNBr-EGF, 4 h; Lane 6. 4.6–42 kDa Ladder; Lane 7. 100% Glyoxyl-EGF, 0 h; Lane 8. 100% Glyoxyl-EGF, 1 h; Lane 9. 86.5% Glyoxyl-EGF, 2 h; Lane 10. 86% Glyoxyl-EGF, 3 h; Lane 11. 87% Glyoxyl-EGF, 4 h. Percentage was referred to the protein found in the supernatant (see “Materials and methods” for details). Quantitative densitometric analysis of the bands was performed with Image Lab software (Fig. S1, Supplementary Information).

According to the results obtained with RNase A, also EGF was immobilized on CNBr-activated-agarose (CNBr-EGF) and provided a quantitative yield (all protein was immobilized). The immobilization yield of EGF on glyoxyl-agarose performed at neutral pH (Glyoxyl-EGF7) and at the same loading was much less (12%). In order to improve the quantity of protein immobilized, EGF was also reacted with glyoxyl-agarose at pH 10.0 (Glyoxyl-EGF10, immobilization yield 29%). Polyacrylamide gel electrophoresis of the supernatant withdrawn during the immobilization was performed to monitor the immobilization reaction (Fig. 4). Lane 1 and 7 show the amount of EGF in the immobilization vessel prior to the start of the immobilization, which can be considered 100% non-immobilized protein. The following samples withdrawn during the immobilization on CNBr activated-agarose did not show any protein in the supernatant (lanes 2–5), thus indicating that all protein was immobilized on the carrier. In the case of EGF immobilization on glyoxyl-agarose (lanes 7–11), the initial amount of protein (100%, lane 1) slightly decreased over time up to 87% residual protein after 4 h, thus accounting for 13% immobilization yield (Fig. 4). In both cases, the results are consistent with the data obtained by the Bradford assay. EGF immobilization on glyoxyl-agarose at pH = 10.0 was also confirmed by gel electrophoresis (data no shown).

### Characterization of immobilized EGF

**Figure 5:**
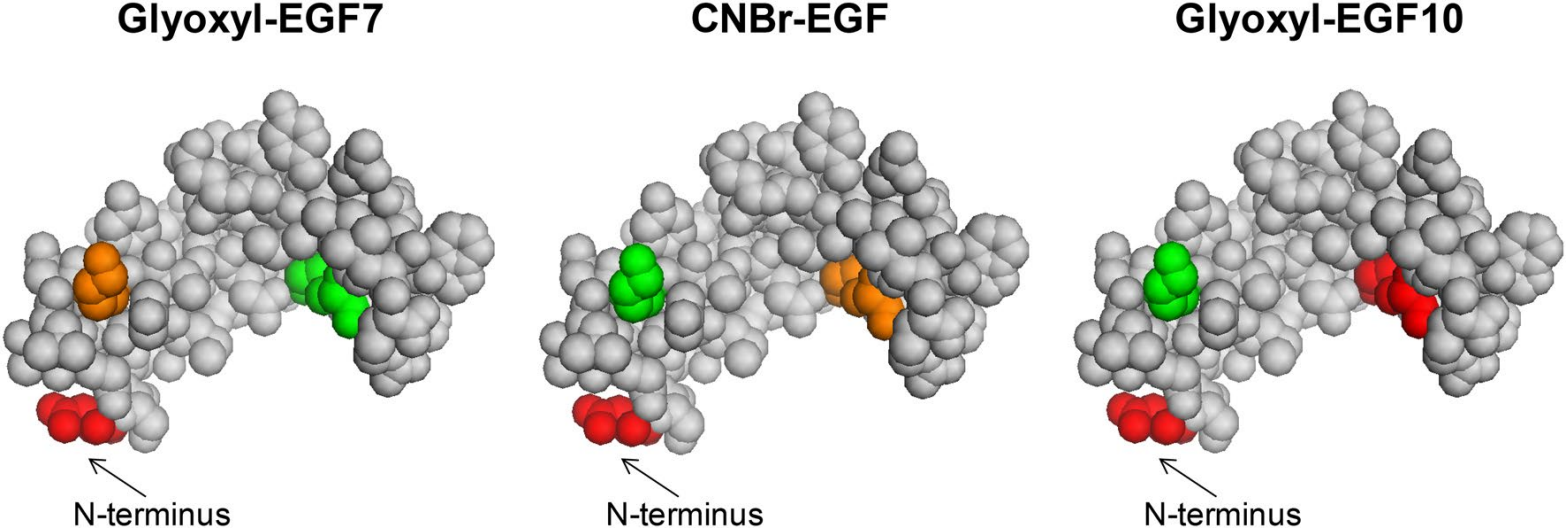
Relative abundance of *N*-terminus and Lys residues obtained by peptide mapping of free amino acids in EGF immobilized on different carriers. Colors indicate the percentage abundance of each residue. The following legend was used: red 0–20%; orange 20–40%; yellow 40–60%; green 60–100%. Consequently, red = Lys bound to the carrier; green = Lys exposed on the surface, not involved in the binding. Created with PyMOL Molecular Graphics System (version 1.1 eval, http://www.pymol.org).

Also EGF bioconjugates were submitted to tryptic digestion in order to assess the involvement of either the two Lys (namely K29 and K49) or the *N*-terminal group in the binding with the carrier. First, soluble EGF was digested under reducing conditions. A sequence coverage of 100% was obtained. The identified peptides are listed in Table S2 (Supplementary Information). The same amount of glyoxyl-agarose-EGF and CNBr-agarose-EGF were thus incubated with chymotrypsin and the resulting peptide analyzed in duplicate by RP-LC–ESI–MS/MS. A significant higher mass area response was obtained for CNBr-agarose-EGF which was considered to reflect the higher immobilization yield than for glyoxyl-agarose. In the LC–MS analysis of both immobilized EGF samples, the peptides containing *N*-terminal position were not detected, differently from soluble EGF, thus suggesting that the two immobilization protocols involve the terminal amino group. Peptides comprising K29 and K49 were identified in both samples. In glyoxyl-agarose-EGF prepared at pH 7 (Glyoxyl-EGF7) the relative abundance of K29 and K49 was comparable to that observed for the soluble peptide (mass area % ratio K49/K29 = 1.76 for soluble peptide and 1.80 for Glyoxyl-EGF7). These data confirm that Lys residues were not involved in the interaction with the carrier. On the other hand, for CNBr-agarose-EGF sample a higher relative abundance of K29 was detected, suggesting a partial involvement of K49 in the binding with the carrier (Table 2, Fig. 5).

**Table 2:** Relative abundance (%) of EGF peptides containing putative binding sites to the agarose carrier, obtained by LC–ESI–MS/MS analysis.

Residue	Soluble EGF (%)	Glyoxyl-EGF7 (%)	CNBr-EGF (%)	Glyoxyl-EGF10 (%)
M1 (*N*-terminal)	23.5	–	–	–
K29	27.7	35.7	69.8	100
K49	48.8	64.3	30.2	–

When Glyoxyl-EGF10 was submitted to chymotrypic digestion, not only *N*-terminal portion, but also the peptides containing K29 were not detectable in LC–MS analysis. This result suggests a two-point immobilization that also involves the more reactive lysine residues, while K29 is not reactive in these conditions.

### In vitro cytotoxicity study

**Figure 6:**
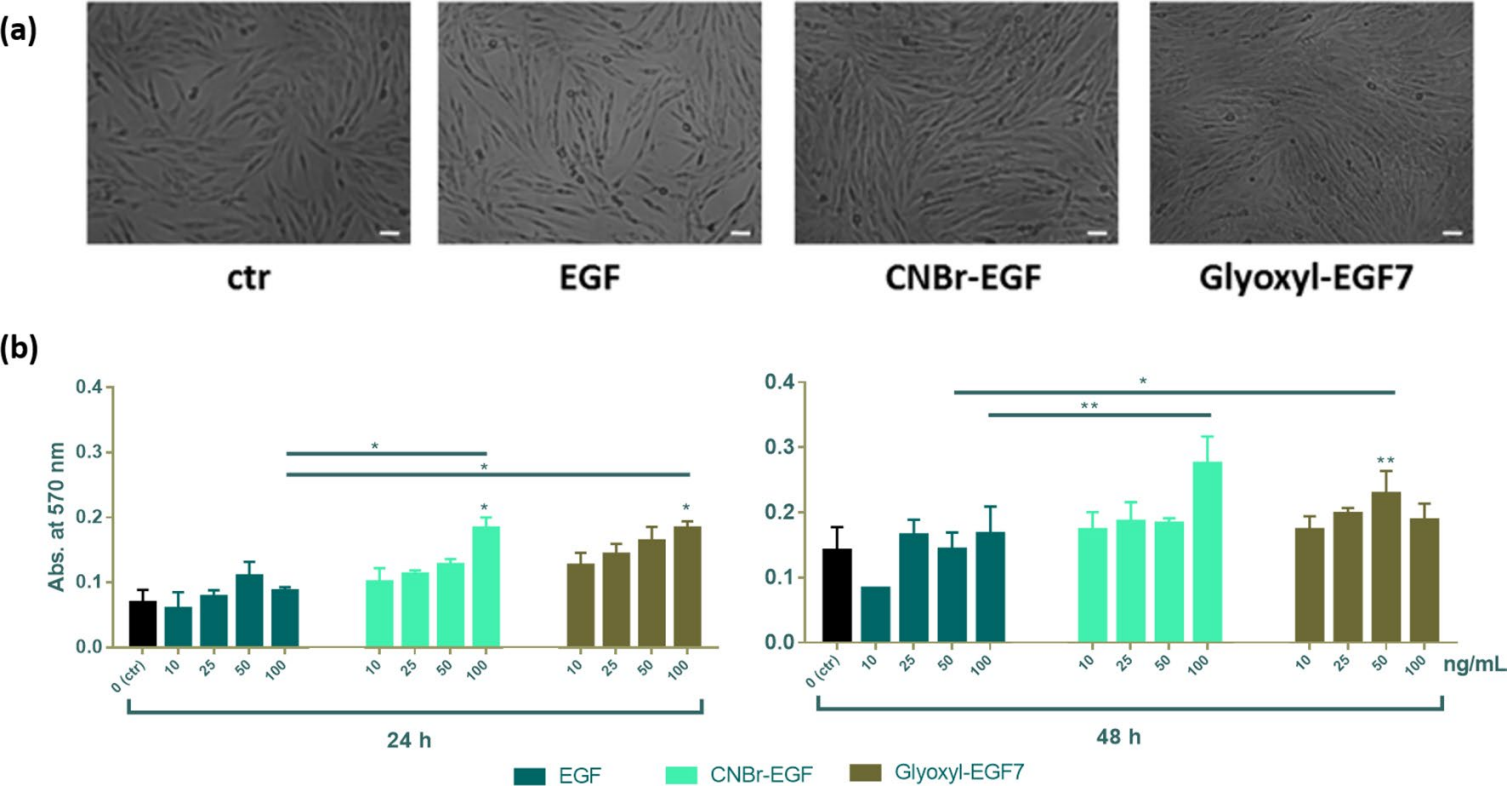
Cytotoxicity activity. (**a**) Characteristics of fibroblast cells, micrographs are shown at × 10 magnification. Scale bar 20 µm. (**b**) In vitro cytotoxicity study on fibroblast cells (10,000 cells/well) at 24, and 48 h. EGF corresponds to soluble epidermal growth factor, CNBr-EGF and Glyoxyl-EGF7 same as EGF immobilized on CNBr-activated-agarose (at pH 7) and glyoxyl-agarose, respectively. Untreated cells have been used as negative control (ctr, 0 ng/mL). A probability value less than 0.05 (*p < 0.05) was defined to be significant and 0.01 (**p < 0.01), 0.001 (***p < 0.001), 0.0001 (****p < 0.0001) as highly significant.

The viability of EGF-carriers treated cells was assessed by the MTT assay. This colorimetric assay is based on the reduction of a yellow tetrazolium salt (3-(4,5-dimethylthiazol-2-yl)-2,5-diphenyltetrazolium bromide or MTT) to purple formazan crystals by metabolically active cells. This assay allows a preliminary assessment of cell viability before performing in-depth in vitro and in vivo-based studies. In vitro cytotoxicity study was evaluated on fibroblast cells (10,000 cells/well) after incubation for 24 and 48 h with soluble EGF, EGF immobilized on CNBr-activated-agarose (CNBr-EGF) and the two derivatives obtained by immobilization on glyoxyl-agarose. The different EGF derivatives were also tested in the same concentration, 10–100 ng/mL. The negative control (ctr −, 0 ng/mL) is represented by untreated cells. As shown in Fig. 6a, no evidence of cytotoxicity was highlighted by microscope analysis after incubation for 48 h at the highest concentration (100 ng/mL). Quantitative analysis revealed a dose-dependent increase in cell in both CNBr-EGF and Glyoxyl-EGF7 over 24, and 48 h (Fig. 6b).

CNBr-EGF (*p < 0.05) and Glyoxyl-EGF7 (*p < 0.05) showed a statically significant increase in absorbance at concentration 100 ng/mL compared to control at 24 h in the same concentration. They also showed significant (p < 0.05) increase in absorbance compared to soluble EGF. After 48 h, Glyoxyl-EGF7 only at 50 ng/mL showed a significant increase compared to control (p < 0.01) and soluble EGF (p < 0.01). There was a significant increase (p < 0.05) in absorbance at 100 ng/mL concentration from free EGF to CNBr-EGF. No significant difference was revealed between CNBr-EGF and Glyoxyl-EGF7 immobilized samples. These two EGF derivatives showed an overall increase in cell viability when compared to the control (CNBr-activated-agarose and glyoxyl-agarose carriers) this finding. The growth observed on the carrier without the immobilized proteins (controls) can be ascribed could be due to agarose acting as a nutrient and/or a scaffold for the cells to grow. However, when the EGF is immobilized on the carrier, the cell growth clearly increase as consequence of the stimulation mediated by the grow factor*.* EGF immobilized on glyoxyl-agarose at pH 10 (Glyoxyl-EGF10) was used as control against its one-point immobilization product (Glyoxyl-EGF7), the data showed an increase in the absorbance at 48 h at each concentration.

**Figure 7:**
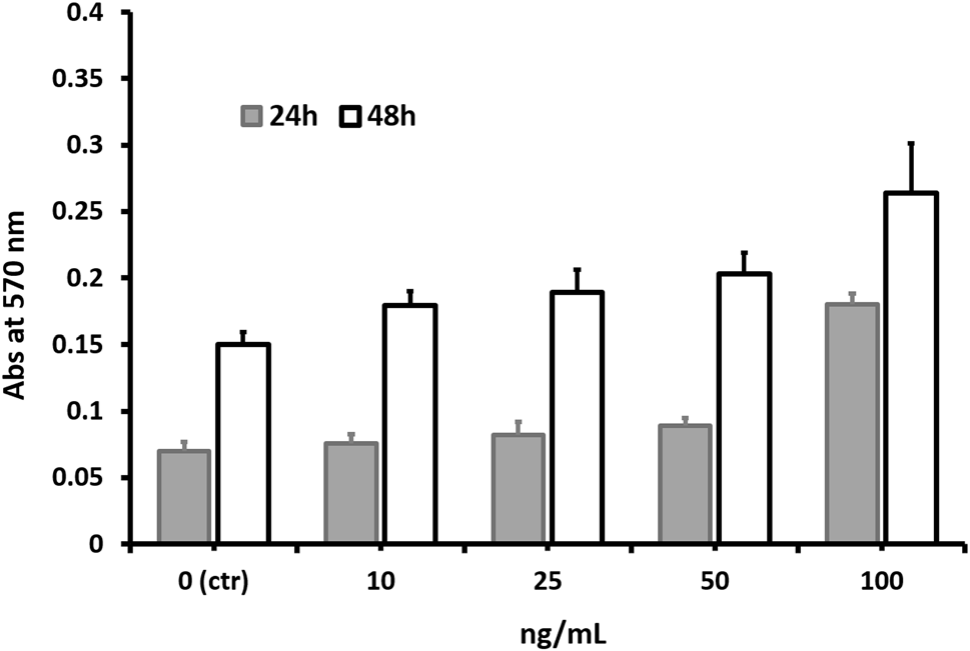
In vitro cytotoxicity study on fibroblast cells (10,000 cells/well) at 24, and 48 h after treatment with Glyoxyl-EGF10 at 10, 25, 50 and 100 ng/mL. Untreated cells have been used as negative control (ctr, 0 ng/mL).

Additionally, the data showed lower values compared with Glyoxyl-EGF7 in particular at 10, 25 and 50 ng/mL (Fig. 7). The reduction of activity observed with Glyoxyl-EGF10 could be ascribed two-point immobilization induced when the process is performed at basic pH, that reduce the freedom and the access at the epitopes responsible of the biological activity compared with the protein immobilized only through the *N*-terminal amino acid.

### Cell viability study

**Figure 8:**
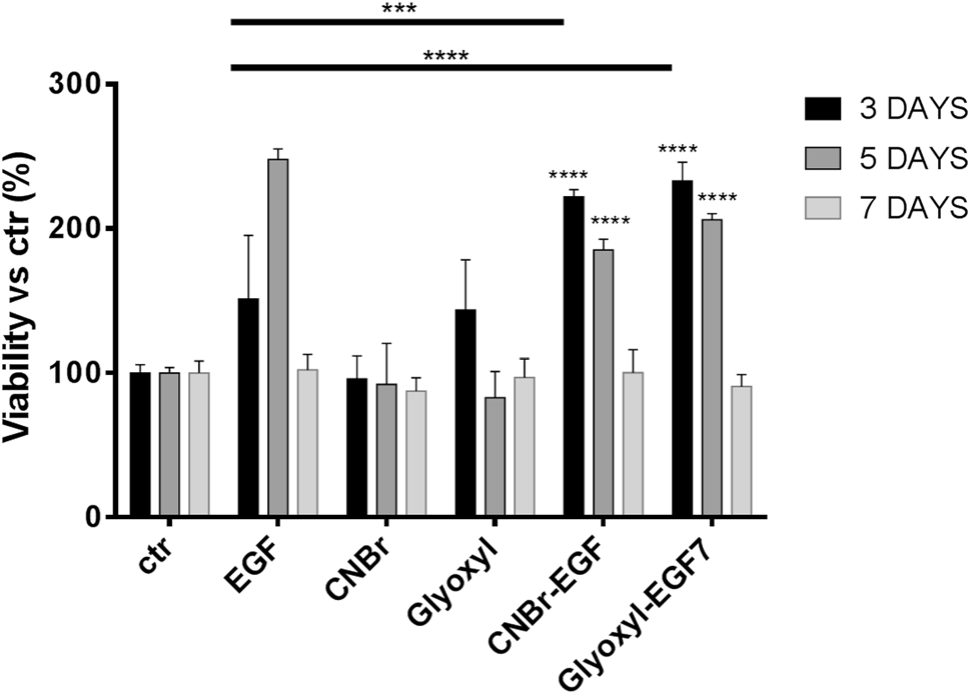
Effect of soluble EGF and immobilized EGF (CNBr-EGF and Glyoxyl-EGF7) on viability of NHDF. Untreated cells (ctr), CNBr-activated-agarose (CNBr) and glyoxyl-agarose (Glyoxyl) carriers were used as controls. Cells were treated for 3, 5 and 7 days with 50 ng/mL of EGF in the soluble form and as immobilized EGF, at day 5 the treatment has been removed and media replaced with fresh complete medium. After an additional incubation for 3 h with 5 mg/mL MTT reagent, the formation of formazan was determined at 540 nm in a photometer plate reader. Data were normalized to average cell viability of CTR (cells w/o treatment). Each measurement was made in triplicate, and data represented the means ± SD. A probability value less than 0.05 (*p < 0.05) was defined to be significant and 0.01 (**p < 0.01), 0.001 (***p < 0.001), 0.0001 (****p < 0.0001) as highly significant by Tukey's multiple comparison test.

The effect of immobilized-EGF on the viability of NHDF has been evaluated by MTT assay with both cells treated with soluble-EGF and immobilized-EGF (CNBr-EGF and Glyoxyl-EGF7), Fig. 8. EGF promoted the viability of fibroblasts, all samples showed a statistically significant increment of cell viability at day 3 and 5 comparing with untreated cells to the same endpoints. CNBr-EGF (*p < 0.05) and Glyoxyl-EGF7 (*p < 0.05) showed a statically significant increase in cell viability at day 3 compared to untreated cells (ctr) in the same concentration. Moreover, the immobilized-EGF samples revealed highly significant increment of cell viability compared to soluble EGF (CNBr-EGF ***p < 0.0001 and Glyoxyl-EGF7 ****p < 0.0001) at the same endpoints. At day 5, cell viability data are consistent with results collected after 3-day incubation, CNBr-EGF and Glyoxyl-EGF7 (****p < 0.0001) presented a statically significant increase in cell viability compared to untreated cells (ctr). Conversely, only CNBr-EGF displayed highly significant enhancement of cell viability with respect to soluble EGF (**p < 0.01). No statistically significant differences have been observed for CNBr- and glyoxyl carriers at all incubation endpoints. At day 7, after treatment removal and culture media renewal, the cell viability percentages went down to 100%. The results were consistent with untreated cells (ctr) and CNBr- and glyoxyl carriers and they can be attributed to the removal of EGF and immobilized-EGF from each culture cells.

### Fluorescent staining and analysis

**Figure 9:**
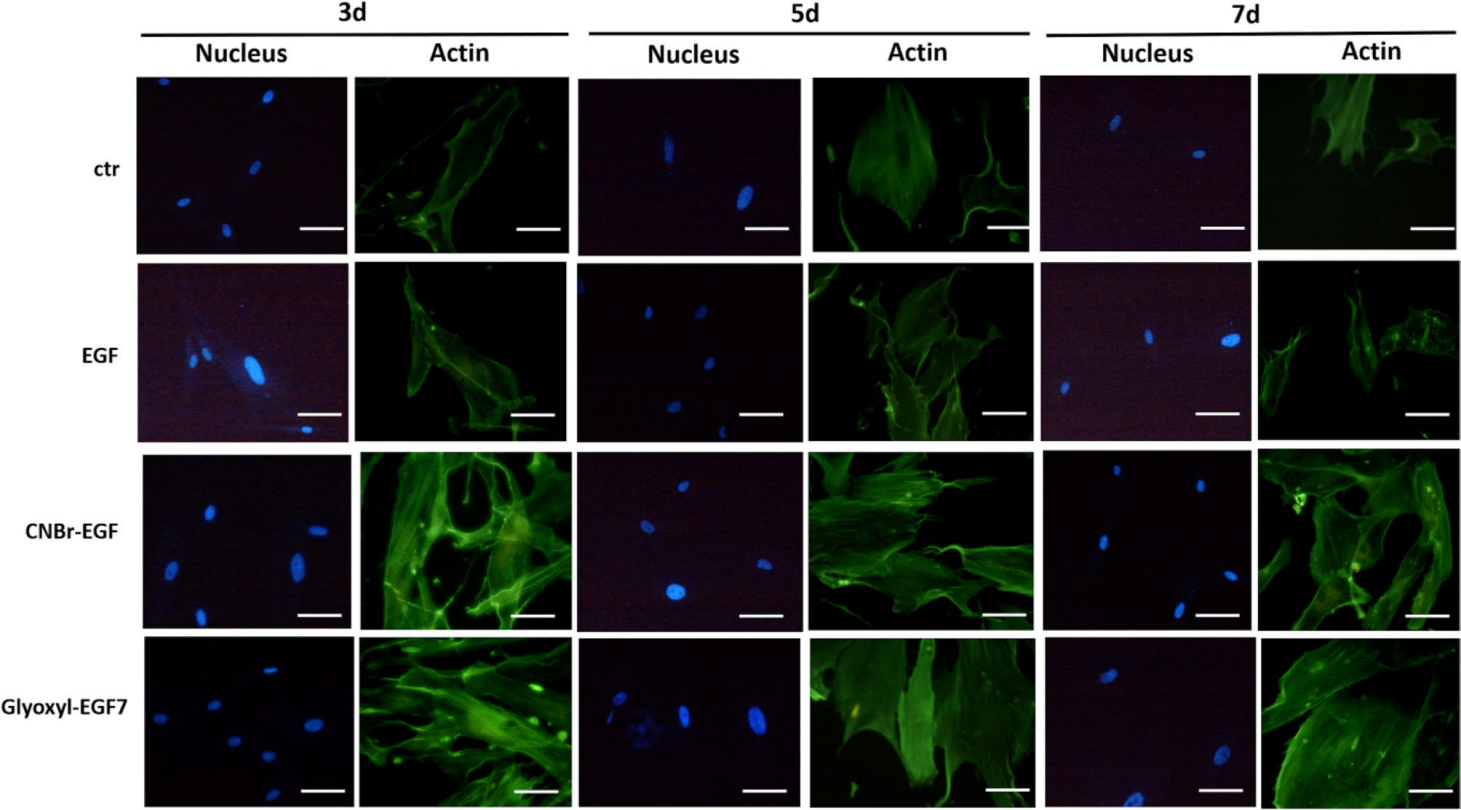
Morphology of NHDF treated with 50 ng/mL soluble-EGF and immobilized-EGF (CNBr-EGF and Glyoxyl-EGF7) incubated for 3, 5 and 7 days. Untreated cells were used as negative control (ctr). After incubation, cells were fixed and stained phalloidin and DAPI. Scale bar, 15 µm.

Fluorescent staining has been exploited to further investigate on interactions of NHDF and their organization after treating with soluble-EGF and immobilized-EGF (CNBr-EGF and Glyoxyl-EGF7). Figure 9 shows representative examples of normal human adult fibroblasts appearance incubated for 3, 5 and 7 days. Cells in basal medium (ctr) were spread with actin stress fibers; a similar behavior has been highlighted for soluble-EGF. The foregoing results show that human fibroblasts behaved in a typical manner when treated with immobilized-EGF and cultured for a similar time. Figure 9 shows the markedly different appearance of fibroblasts, which were largely spread with lamellipodia and extended actin fibers. These data are consistent over all incubation time. These results could be attributed partially to the carrier, it is well demonstrated that the formation of focal adhesions and actin fibers implies that cells growth on a substratum stiff enough to allow isometric tension to develop in the cells. 3D matrix of CNBr-EGF and Glyoxyl-EGF7 carriers seem to influence on cell adhesion and consequently it determines the cellular response. Such influence of 3D matrix on cell behavior is also in agreement with previous studies. Hichem El-Mohri et al*.* demonstrated that fibroblasts displayed differences in the cytoskeleton organization as reflected from enhanced stress fiber formation on stiffer gels. In addition, they proved that the cells spread extensively on stiffer gels as manifested from increased cell area^31,32^.

## Conclusions

The combination of different approaches for protein immobilization and analytical methods for the characterization of immobilized proteins allowed the rational design of active EGF-bioconjugates. The screening of differently functionalized agarose carriers by using RNase A as a “model” protein highlighted that CNBr-agarose can provide a properly oriented protein. Immobilization of EGF on this carrier resulted in a very high immobilization efficiency. From in vitro cytotoxicity assays the immobilized EGF resulted in an enhanced cell viability and show a dose-dependency when seeded with high number of fibroblast cells. Immobilized EGF showed a higher cell proliferative activity compared to soluble EGF, but have similar biological activity compared to each other. The agarose scaffold resulted biocompatible and the product obtained by immobilization of EGF promote cells growth even at high concentrations.

In conclusion, the use of CNBr-activated-agarose seems appropriate for the immobilization of native human EGF driving the process towards the reaction of the *N*-terminal amino acid. In addition, no of reductive steps are required for obtaining stable *C*-*N* bonds (as for example required for immobilization processes mediated by aldehyde groups) ensuring the preservation of the disulfide bonds that stabilize the 3D structure of the protein. Thus, this method could be extended to other growth factors relevant for tissue engineering applications.

## Supplementary Information

Supplementary Information.
