# Supplementary material for: Design of epidermal growth factor immobilization on 3D biocompatible scaffolds to promote tissue repair and regeneration
Source: Sci Rep. 2021 Jan 29;11:2629. doi: 10.1038/s41598-021-81905-1 (PMC7846569; doi:10.1038/s41598-021-81905-1)

SUPPLEMENTARY INFORMATION

Design of Epidermal Growth Factor Immobilization on 3D Biocompatible Scaffolds to Promote Tissue Repair and Regeneration

Teodora Bavaro*, Sara Tengattini, Refaya Rezwan†, Enrica Chiesa, Caterina Temporini, Rossella Dorati, Gabriella Massolini, Bice Conti, Daniela Ubiali and Marco Terreni

Department of Drug Sciences, University of Pavia, viale Taramelli 12, I-27100 Pavia, Italy

†Present address: Department of Pharmacy, ASA University Bangladesh, 23/3 Bir Uttam A.N.M Nuruzzaman Sarak, Dhaka-1207, Bangladesh

*email: teodora.bavaro@unipv.it

Table S1. Detected peptides list after chymotryptic digestion of soluble (***Sol.***) and immobilized (***1-6***) RNAse A samples.

Peptide sequence	***Position***	***RNase A Sample***						
		Sol.	***1***	***2***	***3***	***4***	***5***	***6***
-.KETAAAKF.E	1-8	×	×		×			×
F.ERQHMDSST.S	9-17	×						×
F.ERQHMDSSTSAASSSNY.C	9-25	×	×	×	×	×	×	×
M.MKSRNL.T	30-35	×						
L.TKDRCKPVNTF.V	36-46	×		×			×	×
F.VHESL.A	47-51	×						×
F.VHESLA.D	47-52	×						×
F.VHESLADVQ.A	47-55	×	×	×		×		×
F.VHESLADVQAVC.S	47-58	×	×					×
C.SQKNVACKNGQTNCY.Q	59-73	×	×		×			×
Y.STMSITDCRETGSSKYPNCAY.K	77-97	×						
M.SITDCRETGSSKYPNCAY.K	80-97	×	×					
I.TDCRETGSSKYPNCAY.K	82-97	×						
R.ETGSSKYPNCAY.K	86-97	×						×
Y.KTTQANKH.I	98-105	×						×
Y.KTTQANKHIIV.A	98-108	×						×
Y.KTTQANKHIIVA.C	98-109	×						×
Y.KTTQANKHIIVAC.E	98-110	×						×
Y.KTTQANKHIIVACEGNPY.V	98-115	×	×					
Y.KTTQANKHIIVACEGNPYVPVHF.D	98-120	×						
Q.ANKHIIVAC.E	102-110	×						×
N.KHIIVACEGNPY.V	104-115	×						
C.EGNPYVPVHF.D	111-120	×						×
Y.VPVHF.D	116-120	×	×	×	×	×	×	×
Y.VPVHFDASV.-	116-124	×		×		×		×
V.HFDASV.-	119-124	×						×
H.FDASV.-	120-124	×						×

Table S2. Detected peptides list after chymotryptic digestion of soluble and immobilized EGF.

Peptide sequence	*Position*	*Soluble EGF*	*Immobilized EGF*		
			CNBr	*Glyoxyl pH 7*	*Glyoxyl pH 10*
-.MNSDSECPLSHDGY.C	1-14	×			
-.MNSDSECPLSHDGYCLHDGVCM.Y	1-22	×			
-.MNSDSECPLSHDGYCLHDGVCMY.I	1-23	×			
Y.CLHDGVCM.Y	15-22	×	×	×	×
Y.CLHDGVCMY.I	15-23	×	×	×	×
M.YIEALDKYACNCVVGY.I	23-38	×			
M.YIEALDKY.A	23-30	×	×	×	×
Y.IEALDKY.A	24-30	×	×	×	×
Y.IEALDKYACNCVVGY.I	24-38	×			
Y.IGERCQY.R	39-45	×			
Y.RDLKWW.E	46-51	×	×	×	
Y.RDLKWWELR.-	46-54	×	×	×	
L.KWWELR.-	49-54	×	×	×	

Figure S1. Monitoring EGF immobilization by polyacrylamide gel electrophoresis. Lane 1. 100% CNBr-EGF, 0 h; Lane 2. 0% CNBr-EGF, 1 h; Lane 3. 0% CNBr-EGF, 2 h; Lane 4. 0% CNBr-EGF, 3 h; Lane 5. 0% CNBr-EGF, 4 h; Lane 6. 4.6-42 kDa Ladder; Lane 7. 100% Glyoxyl-EGF, 0 h; Lane 8. 100% Glyoxyl-EGF, 1 h; Lane 9. 86.5% Glyoxyl-EGF, 2 h; Lane 10. 86% Glyoxyl-EGF, 3 h; Lane 11. 87% Glyoxyl-EGF, 4 h. Percentage is referred to the protein found in the supernatant.

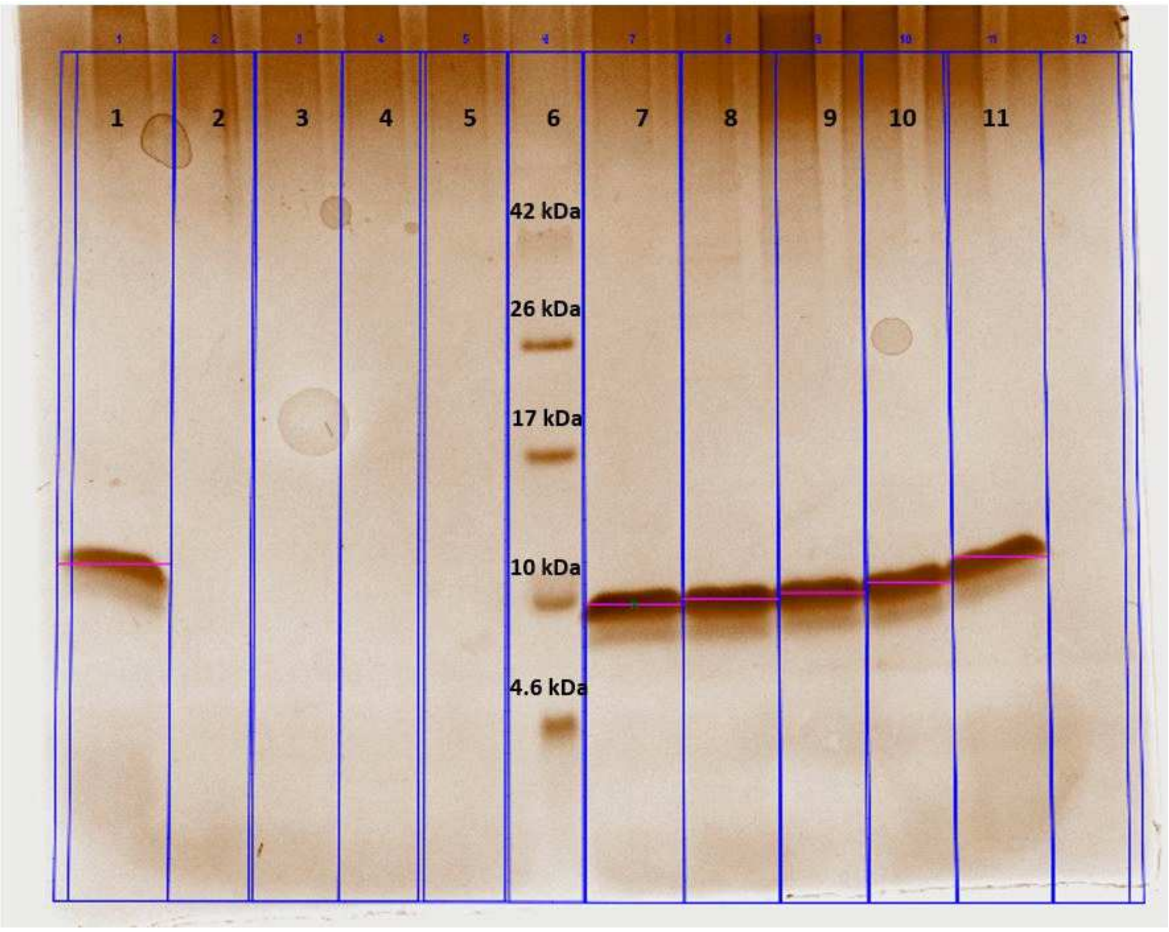

Supplement: Supplementary file 1 — Supplementary Information. [file 41598_2021_81905_MOESM1_ESM.pdf]
